# Supplementary material for: The Devil Lies in the Details: Small Structural and Chemical Changes in Iron Oxide Pigments Largely Alter the Biological Outcomes in Macrophages
Source: Nanomaterials (Basel). 2025 Nov 26;15(23):1772. doi: 10.3390/nano15231772 (PMC12693118; doi:10.3390/nano15231772)
Supplement: Supplementary file 1 [file nanomaterials-15-01772-s001.zip › nanomaterials-3956379-supplementary.pdf]

Control experiment S1: Endotoxin test interference.

In order to test the possible interference of iron oxide pigments with the endotoxin measurement test, the following experiment was performed/

A serial dilution of the endotoxin standard present in the test kit was made, and diluted either with ultrapure water, or with a concentrated pigment solution, so that the end amount of pigment (1.2 mg) was the one present in the 1 ml culture wells in our experiments.

The endotoxin level was then determined through the kit procedure, and the results are displayed in Table S1 (N=2):

Endotoxin concentration (EU/mL)	0	0.125	0.25	1
Control value without pigment				
A	0.040	0.060	0.081	0.376
B	0.045	0.060	0.081	0.381
mean	0.043	0.060	0.081	0.379
value with pigment				
A	0.040	0.066	0.102	0.415
B	0.043	0.065	0.100	0.424
mean	0.041	0.066	0.101	0.420

The results show negligible interference of the pigment with the LAL test (maximum 10%), so that endotoxin levels present in the pigments themselves can be deduced directly from the test without any correction factor.

Control experiment S2: rhodamine 123 test interference

In order to test the possible interference of iron oxide pigments with the rhodamine 123 mitochondrial potential test, the following experiment was performed:

The final rhodamine concentration present in the cell lysate (if 100% of the input were internalized, i.e. 50 µM = 10µM in the cell culture well x5 because of the concentration factor between cell culture well and final lysate) and serial dilutions thereof, was incubated with the final pigment concentration given to the cells in the final cell lysis buffer described in section 2.4, as this is the only moment where the pigment (contained in the lysosomes) and the probe (contained in the mitochondria) could meet. The lysate was then processed according to section 2.4, and its fluorescence read as described in section 2.4. First, an automatic fluorescence gain was used (841) in order to visualize all probes concentrations. Then the same fluorescence gain as the one used in the cellular experiments (1467) leading to saturation of the signal linked to higher rhodamine 123 concentrations and to a signal increase for the lowest one

The results are displayed in Table S2 (N=2):

Rhodamine 123 concentration	0	50 µM	5 µM	0.5 µM
Control value without pigment, gain 841				
A	35	208046	40250	4783
B	36	201728	39306	4876
mean	35.5	204887	39778	4829.5
value with pigment				
A	35	208777	37912	4851
B	37	209175	36519	4902
mean	36	208976	37215.5	4876.5
Control value without pigment, gain 1467				
A	500	**Sat**	**Sat**	143998
B	484	**Sat**	**Sat**	142576
mean	492	**Sat**	**Sat**	143287
A	547	**Sat**	**Sat**	146012
B	596	**Sat**	**Sat**	141147
mean	571.5	**Sat**	**Sat**	143579.5

Sat: detector saturation

The results show negligible interference of the pigment with the Rhodamine uptake test test under all tested conditions. As Rhodamine 123 is also the final product of DHR 123 reoxidation, this control also shows that the final signal of the DHR123 test is free from interference from the pigments

Control experiment S3: DHR test interference.

In order to test the possible interference of iron oxide pigments with the DHR123 oxidative stress test, the following experiment was performed:

The final DHR123 concentration present in the cell lysate (if 100% of the input were internalized, i.e. 500 µM = 100 µM in the cell culture well x5 because of the concentration factor between cell culture well and final lysate) and a 10 fold dilution thereof, was incubated for 30 minutes at room temperature with the final pigment concentration given to the cells in the final cell lysis buffer described in section 2.4. This incubation time was chosen to allow the possible reaction between the pigment and DHR to proceed, while limiting the aerial oxidation of DHR.
The lysate was then processed according to section 2.4, and its fluorescence read as described in section 2.4. First, an automatic fluorescence gain was used (1375) in order to visualize all probes concentrations. Then the same fluorescence gain as the one used in the cellular experiments (1545) leading to saturation of the signal linked to higher rhodamine 123 concentrations and to a signal increase for the lowest one

The results are displayed in Table S2 (N=2):

Rhodamine 123 concentration	0	500 µM	50 µM
Control value without pigment, gain 1375			
A	171	232141	19841
B	251	201779	27137
mean	211	216960	23489
value with pigment			
A	188	200047	22406
B	149	202684	22113
mean	168.5	201365.5	22259.5
Control value without pigment, gain 1545			
A	316	**Sat**	41622
B	504	**Sat**	57634
mean	410	**Sat**	49628
A	351	**Sat**	47568
B	270	**Sat**	46865
mean	310.5	**Sat**	47216.5

Sat: detector saturation

Although the test in this in vitro format is subject to a background aerial oxidation of DHR during the incubation, the results show negligible interference of the pigment with the dihydrorhodamine oxidation test under all tested conditions. In any case, there is no pigment-induced oxidation of the probe.
